# Genetic background affects induced pluripotent stem cell generation

**DOI:** 10.1186/scrt121

**Published:** 2012-08-03

**Authors:** Lauren V Schnabel, Christian M Abratte, John C Schimenti, Teresa L Southard, Lisa A Fortier

**Affiliations:** 1 Department of Clinical Sciences, College of Veterinary Medicine, Cornell University, Ithaca, NY 14853, USA; 2 Department of Biomedical Sciences, College of Veterinary Medicine, Cornell University, Ithaca, NY 14853, USA

## Abstract

**Introduction:**

The influence of genetic background on the ability to generate induced pluripotent stem cells (iPSCs) has the potential to impact future applications, but has yet to be examined in detail. The purpose of this study was to determine if genetic background affects the efficiency of generating iPSCs during early reprograming as well as the pluripotent stability of the iPSCs during later stages of reprograming.

**Methods:**

Mouse embryonic fibroblasts (MEFs) were isolated from six strains of mice (NON/LtJ; C57BL/6J; DBA/2J; BALB/cJ; 129S1/SvlmJ; CAST/EiJ) that were selected based on genetic diversity and differences in ability to produce embryonic stem cell (ESC) lines. MEFs were reprogramed via doxycycline-inducible lentiviral transduction of murine *Oct4*, *Klf4*, *Sox2*, and *c-Myc*. Differences in efficiency to generate iPSCs were assessed by comparing the total number of colonies, the percentage of colonies positive for alkaline phosphatase staining and the percentage of cells positive for SSEA1. iPSC colonies were expanded to establish doxycycline-independent cell lines whose pluripotency was then evaluated via ability to form teratomas in NOD.CB17*-Prkdc^scid^*/J mice. Proliferation of non-transduced parent MEFs from each strain was also examined over ten days under conditions that simulated reprograming.

**Results:**

NON/LtJ and CAST/EiJ strains were more efficient than other strains in generating iPSCs for all parameters measured and parent MEFs from these strains were more proliferative than those from other strains. Doxycycline-independent iPSC lines were established using standard conditions for all strains except BALB/cJ, which required a higher concentration (5x) of leukemia inhibitory factor (LIF). iPSCs from all strains were capable of producing teratomas in NOD.CB17*-Prkdc^scid^*/J mice.

**Conclusions:**

The results of this study suggest that genetic background does affect iPSC generation and pluripotent stability. In addition, our results demonstrate that strain differences in efficiency to generate iPSCs during the early stages of reprograming are correlated with those observed in proliferation of parent MEFs. These findings have important implications both for future iPSC applications as well as for future investigation into determining the genes responsible for reprograming efficiency and stability.

## Introduction

The induced pluripotent stem cell (iPSC) field continues to make rapid advances in terms of optimizing reprograming methods to circumvent clinical safety issues and characterization of the genetic and epigenetic composition of established iPSC lines [1-4]. The influence of genetic background on the ability to generate iPSCs, as well as the stability and quality of derived iPSCs for downstream applications, also has the potential to impact the future applications. However, the role of genetic background has yet to be examined in significant detail. The effect of genetic background on pluripotency has precedence in mice; it is well documented that there are dramatic strain differences in ability to produce embryonic stem cell (ESC) lines [5-8].

Many of the mouse iPSC studies to date have used mouse embryonic fibroblasts (MEFs) from transgenic mice of an undefined or hybrid background [9], or have used MEFs or tail tip fibroblasts (TTFs) derived from animals originally produced from hybrid ESCs [9-16]. Few studies have used MEFs or TTFs from a pure inbred strain [17-19]. To our knowledge, only one study to date has directly compared the ability of two different inbred strains to generate iPSCs [17]. In this study, Hanna *et al*. found that MEFs from NOD/ShiLtJ mice, a strain previously considered nonpermissive for ESC derivation, were capable of generating iPSCs, but that these iPSCs were dependent on exogenous transgene expression unlike the iPSCs derived from control 129Sv/Jae MEFs [17]. The authors determined that the NOD/ShiLtJ iPSCs were dependent upon ectopic expression of either KLF4 or c-MYC using constitutive lentiviruses, and that the cells were able to overcome this factor dependence when cultured in media supplemented with any of the following proteins or small molecules: WNT3a, which promotes iPSC derivation in the absence of c-MYC [20]; CHIR99021, a GSK3b inhibitor; or Kenpaullone, a GSK3b and CDK1/cyclin B inhibitor which has been shown to replace KLF4 during iPSC reprograming [17,21]. As the authors concluded, these results suggest that genetic background can affect the pluripotent stability of iPSCs and that reprograming and culture conditions may have to be modified for certain strains [17].

**Figure 1:**
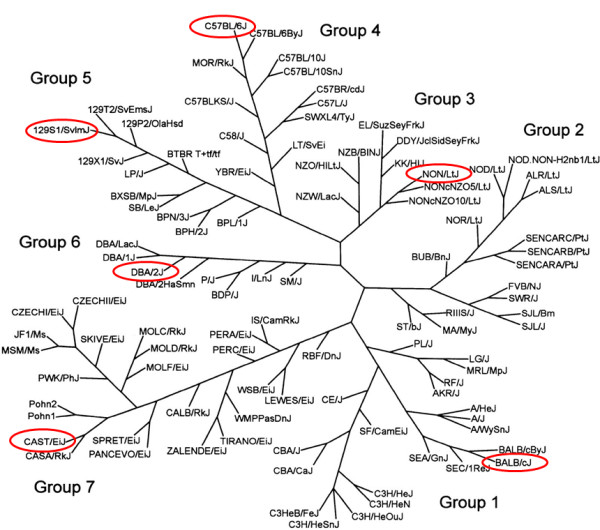
**Mouse family tree**. The seven mouse groups as described as by Petkov *et al*. [22] with the strains examined in this study circled in red. Group 1, Bagg albino derivatives; Group 2, Swiss mice; Group 3, Japanese and New Zealand inbred strains; Group 4, C57/58 strains; Group 5, Castle's mice; Group 6, CC Little's DBA and related strains; Group 7, wild-derived strains. Modified from Petkov *et al*. and reprinted with permission [22]. The length and angle of the branches were optimized for printing and do not reflect the actual evolutionary distances between strains.

The purpose of this study was to determine if genetic background affects the efficiency of generating iPSCs during early reprograming as well as the pluripotent stability of the iPSCs during later stages of reprograming. We chose six different inbred strains of mice to examine, based on their genetic diversity [22-24] and on their differences in ability to produce ESC lines [5-8]. These six strains included five classical laboratory strains (NON/LtJ, C57BL/6J, DBA/2J, BALB/cJ, and 129S1/SvlmJ) and one wild-derived inbred strain (CAST/EiJ) (Figure 1). Because 129-derived substrains such as 129S1/SvlmJ support facile ESC line derivation [5-8], while both C57BL/6J and BALB/cJ mice do not [5,8], we reasoned that these strains would be useful for assessing potential differences in reprograming efficiency. In addition, three of the strains (C57BL/6J, 129S1/SvlmJ, and CAST/EiJ) are progenitors of the Collaborative Cross that is proving effective for analyzing complex genetic phenotypes [25,26]. Knowledge on the potential differences between these strains in their ability to generate iPSCs and their pluripotent stability might therefore be amenable to genetic analysis.

In this study, we show that iPSC lines can be generated from all six of the strains examined using a lentiviral reprograming system [27-29] and that these cell lines are capable of forming teratomas in NOD.CB17*-Prkdc^scid^*/J mice. We demonstrate, however, that there are strain differences in efficiency of iPSC induction, growth, and maintenance requirements, and that these strain differences correlate with proliferative ability of the parental MEFs.

## Materials and methods

### Mice

Male and female mice from all six strains examined (NON/LtJ; C57BL/6J; DBA/2J; BALB/cJ; 129S1/SvlmJ; CAST/EiJ ) were purchased from The Jackson Laboratory (Bar Harbor, ME, USA). For each strain, breeder trios were established for timed matings such that embryonic day-13.5 embryos could be collected and processed to generate MEFs. NOD.CB17*-Prkdc^scid^*/J mice, used for teratoma formation assays, were also purchased from The Jackson Laboratory. The use of mice in this study was approved by the Institutional Animal Care and Use Committee of Cornell University.

### MEF culture

Embryonic day-13.5 embryos were isolated from the uteri of pregnant mice, lavaged with phosphate buffered saline (PBS), and eviscerated. Each embryo was then gently homogenized in MEF media comprising high glucose Dulbecco's modified eagle's medium (DMEM) containing 10% fetal bovine serum (FBS), penicillin (100 units/mL), and streptomycin (100 units/mL) and the resultant cell suspension was transferred to a 100-mm tissue culture plate and incubated at 5% CO_2_, 90% humidity, and 37°C. The plates were washed with PBS and the media changed daily until the MEFs were confluent, at which time they were trypsinized, resuspended in freeze media comprising DMEM with 10% FBS and 10% dimethyl sulfoxide (DMSO), and cryopreserved until further use. All experiments were performed using MEFs derived from two different embryos for each strain.

### Lentiviral constructs

Lentiviral vectors for doxycycline-inducible transgene expression were constructed as previously described [27-29] using an FUW-based plasmid with a tetracycline operator (TetO) and a constitutive cytomegalovirus (CMV) promoter. Briefly, the viral packaging plasmids psPAX2 and pMD2.G (Addgene 12260 and 12259, Cambridge, MA, USA) as well as the plasmids encoding the reverse tetracycline transactivator (M2rtTA; Addgene 20342, Cambridge, MA, USA) and the mouse factors *Oct4*, *Sox2*, *Klf4 *and *c-Myc *(Addgene 20323, 20326, 20322 and 20324 respectively, also Cambridge) were purified from bacterial cultures. The vectors were then prepared by co-transfecting the viral packaging plasmids with plasmids encoding the reverse tetracycline transactivator and the reprograming factors into 293T cells using the FuGENE^®^6 Transfection Reagent (Roche Applied Science, Indianapolis, IN, USA). Viral supernatants were collected at 48 and 72 hours, concentrated using an Amicon Ultra-15 centrifugal filter unit with an Ultracel-30 membrane (Millipore, Billerica, MA, USA), filtered through a 0.45 um filter, and stored in liquid nitrogen until used.

### Reprograming of MEFs and iPSC culture

Passage 2 (P2) MEFs from each strain were seeded onto gelatin-coated tissue culture plates at a density of 6.75 × 10^3 ^cells/cm^2 ^in MEF media and allowed to adhere for 24 hours [27]. The culture media was then replaced with fresh MEF media supplemented with the viral supernatant described above. Following 24 hours of incubation with the viral supernatant, the culture media was changed to ESC media (KnockOut™ DMEM (Gibco, Grand Island, NY, USA) supplemented with 15% KnockOut™ Serum Replacement (Gibco), recombinant LIF, MEM non-essential amino acids solution (100 μm), 2 mM GlutaMAX™(Gibco), 0.1 mM 2-mercaptoethanol, penicillin (100 units/mL), streptomycin (100 units/mL), and doxycycline (2 μg/mL; Sigma, St. Louis, MO, USA)). Cells destined for flow cytometric analysis and for expansion were kept on original 6-well plates while cells destined for alkaline phosphatase (AP) staining and colony counting were trypsinized and passaged onto gelatin-coated 60 mm tissue culture plates seeded with feeder cells (Cs irradiated C57BL/6J × 129S1/SvImJ1 MEFs) prior to the start of reprograming with doxycycline. For all plates, ESC media was refreshed daily during reprograming

### AP staining and colony counting

AP staining was performed directly on the 60-mm plates using the Vector Red Alkaline Phosphatase Substrate Kit (Vector Laboratories, Burlingame, CA, USA) according to the manufacturer's directions. Both the number of AP-stained colonies and the total number of colonies on the plates were quantified using bright field microscopy at 100× magnification. Colonies were identified based on the following morphological criteria: well defined-border, three-dimensionality, and tightly packed cells. A grid system was used on the plates to facilitate colony counting. Each plate was counted twice and the mean number of AP-stained colonies and the mean total number of colonies was determined. The percentage of AP stained colonies was determined by dividing the mean number of AP-stained colonies by the mean total number of colonies and then multiplying by one hundred.

### Flow cytometric analysis

Cells from the 6-well primary transformation plates were trypsinized, washed with PBS, fixed in 4% paraformaldehyde, washed again, and resuspended in blocking buffer (TBS buffer, 0.1% Triton X-100, and 1% BSA) overnight at 4°C. The cell pellet was then washed, resuspended in unconjugated primary antibody for 1 hour at 4°C, washed, and resuspended in a secondary fluorescent-conjugated antibody for an additional 1 hour at 4°C. Cells were resuspended in blocking buffer and analyzed on a BD LSR II (Becton Dickinson Immunocytometry Systems, San Jose, CA, USA) flow cytometer and FACSDiva software (Becton Dickinson). Data were collected on 1 × 10^4 ^cells. Double staining with primary antibodies against SSEA1 (Millipore MAB4301, Billerica, MA, USA) and LIN28 (Abcam Inc. ab46020, Cambridge, MA, USA) with respective fluorescein isothiocyanate (FITC) (SouthernBiotech 1010-02, Birmingham, AL, USA) and PerCP-Cy5.5 (Santa Cruz Biotechnologies sc-45101, Santa Cruz, CA, USA) conjugated secondary antibodies was performed with resultant quadrant statistics including percentage of positive cells in each quadrant. Calibration of the flow cytometer and setting of gates was performed using non-transduced P2 MEFs as negative controls and established 1-A4 (C57BL/6J ×129S1/SvlmJ) iPSCs and v6.4 (C57BL/6J × 129S4/SvJae) ESCs (530 You,Y. 1998) as positive controls. The 1-A4 iPSC line was generated in our laboratory and validated via teratoma formation in NOD.CB17*-Prkdc^scid^*/J mice and ability to generate germline chimeras through blastocyst injection.

### iPSC line generation

iPSC colonies from 6-well primary transformation plates were picked with pipette tips into individual wells of 96-well tissue culture plates containing trypsin. The trypsin was neutralized with DMEM and 10% FBS, and the cells within each well were then transferred to individual wells of 96-well tissue culture plates seeded with feeder cells in ESC media and expanded. Doxycycline was removed from the media at the 6-well plate stage (around P7) in order to establish doxycycline-independent cell lines from each strain. The cells were then further expanded (P10 to P15) in order to reach the cell numbers necessary for teratoma formation assays and for cryopreservation of stock from each strain.

### Teratoma formation and histological analysis

iPSCs from one doxycycline-independent cell line from each strain were trypsinized, pelleted and suspended at 1 × 10^7 ^cells/mL in MEF media, then 150μl of the cell suspension (1.5 × 10^6 ^cells) was injected subcutaneously into the flank of a NOD.CB17*-Prkdc^scid^*/J mouse. For each strain, six injections were performed in 3 NOD.CB17*-Prkdc^scid^*/J mice (both flanks of each mouse were injected). Four to five weeks post-injection, tumors were surgically dissected, fixed in 4% paraformaldehyde, embedded in paraffin, sectioned, and stained with hematoxylin and eosin. All histologic sections were reviewed by a board-certified veterinary pathologist (TLS.) for teratoma formation.

### MEF proliferation assays

Proliferation of non-transduced parent P2 MEFs from each strain was examined every 2 days over a total of 10 days. We seeded 1.9 × 10^5 ^MEFs on each 60 mm tissue culture plate to be cultured and later harvested at the indicated time points to perform cell counts. MEFs were maintained in standard MEF media for the first 24 hours and then the media was changed to ESC media supplemented with doxycycline to simulate reprograming conditions for the remainder of the assay. Assays were performed using MEFs derived from two different embryos for each strain.

## Results and discussion

### Strain differences in efficiency to generate iPSCs are manifested in the early stages of reprograming

**Figure 2:**
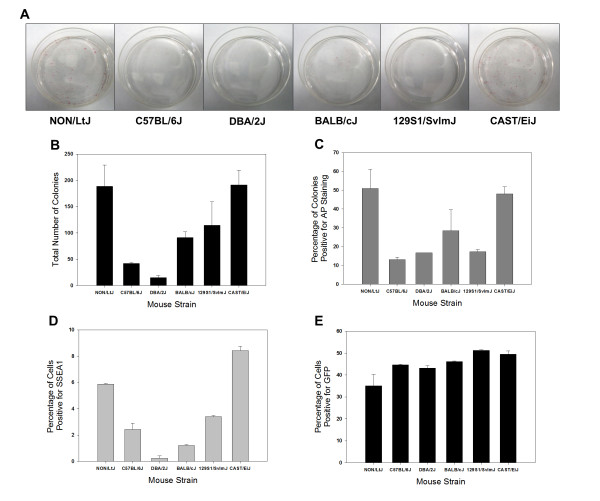
**Strain differences during early iPSC reprograming**. Gross strain differences in efficiency to generate induced pluripotent stem cell (iPSC) colonies were observed after 8 days of mouse embryonic fibroblast (MEF) reprograming as visualized in these photographs with alkaline phosphatase (AP) staining (**A**). Strain differences were quantified by total number of colonies (**B**), percentage of colonies positive for alkaline phosphatase staining (**C**) and percentage of cells positive for SSEA-1 (**D**). NON/LtJ and CAST/EiJ strains were consistently more efficient than other strains in generating early iPSCs. These differences were not believed to be due to strain differences in MEF transducibility, as demonstrated by the percentage of cells positive for green fluorescent protein (GFP) on day 8 following transduction with a lentiviral GFP vector (**E**). Experiments were performed using MEFs derived from 2 different embryos for each strain (data presented as mean ± SD).

In order to assess potential strain background effects on iPSC generation during early reprograming, the primary transformation and 60 mm plates were evaluated for the total number of colonies, the percentage of colonies positive for AP staining, and the percentage of cells positive for SSEA1 and LIN28 expression. On both the 60-mm plates in which the cells were used for AP staining and colony counting and the 6-well primary transformation plates in which the cells were used for flow cytometric analysis, gross differences in the generation of iPSC colonies were observed such that cells had to be stained and counted, or harvested for flow cytometry, after only 8 days of reprograming in order to avoid overconfluency of cells from the most efficient strains (Figure 2A). This time point was much earlier than expected based on the doxycycline-inducible lentiviral reprograming system literature in which colonies are generally passaged or picked off of primary transformation plates around 13 to 21 days for expansion and/or evaluation [27-29], and stresses the differences that can be observed when using strains of diverse genetic backgrounds. Because the iPSCs were harvested at this very early time point of 8 days, the resultant LIN28 expression was negative in the iPSCs from all six strains and only SSEA1 expression was included in the final analysis. This finding is consistent with the literature in which LIN28 is used as a marker for more established iPSCs and ESCs [30-32] as confirmed by our control iPSC (1-A4) and ESC (v6.4) lines.

During early reprograming, MEFs from all of the strains formed cell colonies exhibiting typical iPSC morphology that were AP-positive within 8 days after the start of reprograming. However, the total number of colonies and the percentage of AP-positive colonies varied dramatically between the strains. Notably, NON/LtJ and CAST/EiJ strains were more efficient than other strains (Figures 2B and 2C). Similarly, the percentage of cells positive for SSEA1 varied between the strains but paralleled the previous two parameters with NON/LtJ and CAST/EiJ having the highest percentage (Figure 2D). As expected, the percentage of cells positive for SSEA1 was low (between 0.11 and 8.64%) at this early time point of 8 days after the start of reprograming for all strains. Using the same doxycycline-inducible lentiviral reprograming system, Brambrink *et al*. previously demonstrated that SSEA1 expression appears between 3 and 9 days of reprograming, whereas AP activity appears within 3 days of reprograming [27]. Brambrink *et al*. also showed that after 9 days of reprograming, about 7% of AP-positive cells were also SSEA1-positive [27]. This percentage of SSEA1-positive cells is consistent with our findings.

To ensure that the differences amongst strains in reprograming efficiency were not due to differences in lentiviral infection, P2 MEFs were seeded on 6-well plates at the same density as they were for reprograming, transduced with a lentiviral green fluorescent protein (GFP) vector (Addgene 14883, Cambridge, MA, USA) and maintained under reprograming conditions. After 8 days, the cells were trypsinized and the percentage of GFP-positive cells was determined using flow cytometry. The percentage of GFP-positive cells was very similar for all strains, ranging from 35.10 ± 5.23% (mean ± SD) for NON/LtJ MEFs to 51.25 ± 0.21% for 129S1/SvImJ1 MEFs, suggesting that the strain differences in efficiency to generate iPSCs were not due to strain differences in MEF transducibilty (Figure 2E).

### Differences in proliferation of parent non-transduced MEFs correlate with differences in efficiency to generate iPSCs during early reprograming

**Figure 3:**
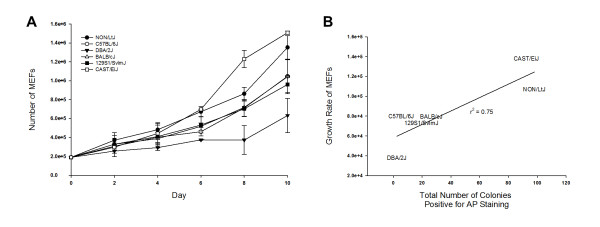
**Strain differences in mouse embryonic fibroblast (MEF) proliferation**. Strain differences in MEF proliferation were observed over 10 days and were well correlated with the observed strain differences in efficiency to generate induced pluripotent stem cells (iPSCs) during early reprograming. Non-transduced parent P3 MEFs from each strain were seeded at 1.9 × 10^5 ^cells per 60-mm tissue culture plate on day 0 and then counted every 2 days over a total of 10 days (**A**). MEFs were maintained in standard MEF media for the first 24 hours after which the media was changed to embryonic stem cell (ESC) media supplemented with doxycycline to simulate reprograming conditions. MEFs derived from two different embryos were evaluated for each strain (data presented as mean ± SD). The growth rate of the MEFs from each strain was determined from the slope of the linear regression curve fitted to the data set in (**A**) for each strain. The growth rate of each strain was then plotted against the total number of colonies positive for alkaline phosphatase (AP) staining and a line of best fit determined, revealing a moderately strong positive correlation between iPSC generation efficiency and MEF proliferation as indicated by the r^2 ^value (**B**).

Proliferation of non-transduced parent P2 MEFs was examined every 2 days over a total of 10 days in order to determine if genetic differences in MEF proliferation could potentially be affecting the efficiency of iPSC generation (Figure 3A). Strain differences in MEF proliferation were observed over the 10 day period and a positive correlation was found between MEF growth rate and efficiency to generate iPSCs during early reprograming. This is demonstrated in Figure 3B where MEF growth rate and total number of colonies positive for AP staining are compared with a resultant r^2 ^value of 0.75. In particular, NON/LtJ and CAST/EiJ MEFs were the most proliferative and most efficient in generating iPSCs while DBA/2J MEFs were the least.

Interestingly, fibroblasts capable of increased proliferation through *Trp53 *deletion have increased iPSC generation efficiency [33-35]. It is possible that MEFs of the most efficient strains found in this study, NON/LtJ and CAST/EiJ, have a reduced rate of senescence compared to the other strains, which is allowing for more effective reprograming. The fact that the most proliferative MEFs were of the CAST/EiJ strain is also of interest as this wild-derived inbred strain is the most genetically distinct strain that we examined.

The finding of this study that cellular proliferation rate is correlated with iPSC generation efficiency is consistent with those of Ruiz *et al*. in which the induction of cellular proliferation (through downregulation of pRb) increased human iPSC reprograming efficiency [36]. In that study, Ruiz *et al*. also elegantly demonstrated that cell cycle arrest (through induction of the arrest inducers p15, p16, or p21) inhibits reprograming and actually drives iPSCs towards irreversible differentiation [36]. A potential follow-up study to this one in order to further elucidate the mechanisms behind the differences in genetic background effects on iPSC efficiency would be to alter the cellular proliferation of the MEFs for each strain, either through induction or arrest, and then examine the iPSC generation efficiency.

### BALB/cJ iPSCs require a higher concentration of LIF than other strains for cell line expansion and doxycycline independence

In order to determine if genetic background affects the pluripotent stability of iPSCs during later stages of reprograming, iPSC lines from all six strains were established and further expanded without doxycycline supplementation. Doxycycline-independent iPSC lines could be established using our standard conditions and ESC media for all strains except BALB/cJ, which were established only when supplemented with a higher concentration (5×) of leukemia inhibitory factor (LIF). This finding suggests that BALB/cJ iPSCs may have reduced pluripotent stability and is consistent with the BALB/cJ ESC literature in which BALB/cJ ESC lines were established only when using a 5× higher concentration of LIF than that needed for other strains [5-7]. The mechanism behind this requirement for increased LIF supplementation in BALB/cJ cells has yet to be identified.

### Doxycycline-independent cell lines from all strains are capable of producing teratomas in SCID mice

**Figure 4:**
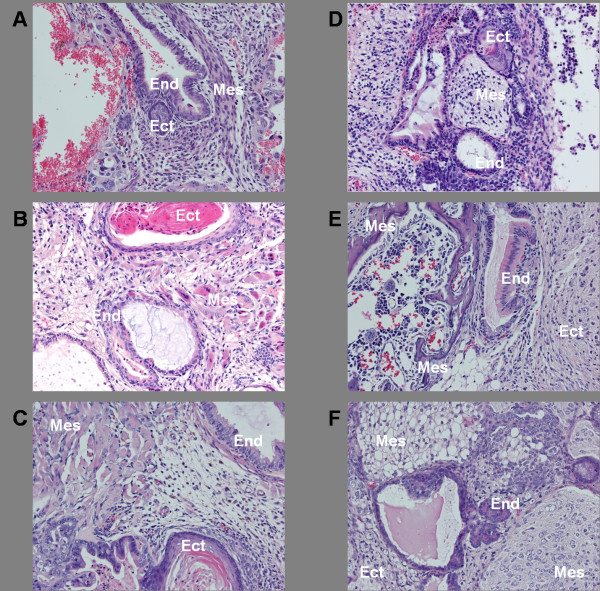
**Pluripotency of induced pluripotent stem cells (iPSCs) derived from mouse embryonic fibroblast (MEFs) of each strain**. Doxycycline-independent cell lines from all the strains were capable of producing teratomas in NOD SCID mice by 5 weeks post-injection as shown in these histologic images, all of which are stained with hematoxylin and eosin and viewed at 200× magnification. (**A**) NON/LtJ; (**B**) C57BL/6J; (**C**) BA/2J; (**D**) BALB/cJ; (**E**) 129S1/SvlmJ; (**F**) CAST/EiJ. Tissues from all three germ layers were identified on each section as indicated by the labels: Ect, ectoderm; Mes, mesoderm; End, endoderm.

Doxycycline-independent cell lines from all the strains were capable of producing teratomas in NOD.CB17*-Prkdc^scid^*/J mice by 5 weeks post injection, thereby demonstrating pluripotency (Figure 4). For all strains, the cell lines were between P10 and P15 and were the initial cell lines chosen for the teratoma assay. None of the cell lines from any of the strains required a repeat set of injections or the assay to be repeated with a different cell line.

iPSC lines in this study were not evaluated for their ability to generate chimeras or for germline competence, making direct comparison to the ESC literature on the effect of genetic background difficult beyond the finding of reduced pluripotent stability already discussed for the BALB/cJ strain. While the 129S1/SvlmJ strain was moderately efficient in generating IPSCs compared to the other strains in this study and the 129S1/SvlmJ iPSC line was readily able to form teratomas in NOD.CB17*-Prkdc^scid^*/J mice, a conclusion cannot be drawn from these data as to whether or not this strain is as useful for generating iPSCs as it has been shown to be for generating ESCs for transgenic applications [6,7]. In addition, the two most efficient strains in this study, NON/LtJ and CAST/EiJ, are strains that have not been examined for their ability to generate ESCs, making them intriguing candidates for future studies.

## Conclusions

Our comparison of six different inbred mouse strains has revealed that genetic background does affect both the efficiency of generating iPSCs during the early stages of reprograming as well as the pluripotent stability of the cells during later stages of reprograming. These findings suggest that genetic background must be considered when interpreting results of iPSC studies in the literature and that iPSC derivation may need to be customized for different strains. In addition, our findings suggest that the proliferation rate of the fibroblasts is positively correlated with iPSC generation, suggesting a possible simple laboratory screening parameter to predict iPSC generation efficiency.

The two most efficient strains in this study, NON/LtJ and CAST/EiJ, may prove useful in the future for deriving iPSCs for transgenic purposes, as iPSCs from these strains appear to be robust. One limitation to this study, however, was that we did not evaluate the iPSCs lines for their ability to generate chimeras and for germline competence. This information is essential for ultimately determining which strain may be most beneficial for transgenic applications.

In conclusion, we have shown that there are strain differences in efficiency to generate iPSCs during the early stages of reprograming and that these strain differences are correlated with those observed in proliferation of parent MEFs. We have also shown that there are strain differences in pluripotent stability as far as ability to expand iPSC lines and achieve doxycycline independence. These findings have important implications both for future iPSC applications as well as for future investigation into determining the genes responsible for reprograming efficiency and stability. It is possible that the Collaborative Cross, of which three of the strains examined in this study are progenitors, could be used to identify such genes.

## Abbreviations

AP: alkaline phosphatase; BSA: bovine serum albumin; CMV: cytomegalovirus; DMEM: Dulbecco's modified eagle's medium; DMSO: dimethyl sulfoxide; ESC: embryonic stem cell; FITC: fluorescein isothiocyanate; GFP: green fluorescent protein; iPSC: induced pluripotent stem cell; LIF: leukemia inhibitory factor; MEF: mouse embryonic fibroblast; P2: passage 2; PBS: phosphate buffered saline; SSEA1: stage-specific embryonic antigen 1; TetO: tetracycline operator; TTF: tail tip fibroblast.

## Competing interests

The authors declare that they have no competing interests.

## Authors' contributions

LVS, CMA, JCS, and LAF designed the study. LVS and CMA performed the experiments. LVS performed tissue collections (for teratoma assays), on which LVS and TLS carried out histologic assessments. All authors contributed to data analysis and interpretation. LVS, JCS, and LAF were responsible for drafting the manuscript. All authors revised the manuscript and approved the final version.

## Authors' information

Author details

1) Lauren V Schnabel, VMC C3-105, College of Veterinary Medicine, Cornell University, Ithaca, NY 14853, USA. Email: lvs3@cornell.edu

2) Christian M Abratte, VRT T9-010, College of Veterinary Medicine, Cornell University, Ithaca, NY 14853, USA. Email: ca258@cornell.edu

3) John C Schimenti, VRT T9-014A, College of Veterinary Medicine, Cornell University, Ithaca, NY 14853, USA. Email: jcs92@cornell.edu

4) Teresa L Southard, Diagnostic Lab North, Room A1-212, Cornell University, Ithaca, NY 14853, USA. Email: tls93@cornell.edu

5) Lisa A Fortier, VMC C3-181, College of Veterinary Medicine, Cornell University, Ithaca, NY 14853, USA. Email: laf4@cornell.edu. §Corresponding author.
